# Measuring serum concentrations of interleukin-33 in atopic dermatitis is associated with potential false positive results

**DOI:** 10.1186/s40064-016-1673-z

**Published:** 2016-01-13

**Authors:** Uffe Nygaard, Christian Vestergaard, Claus Johansen, Mette Deleuran, Malene Hvid

**Affiliations:** Department of Dermatology and Venereology, Aarhus University Hospital, P.P. Ørumsgade 11, 8000 Århus C, Denmark; Department of Clinical Medicine, Aarhus University, 8000 Århus C, Denmark; Department of Biomedicine, Aarhus University, 8000 Århus C, Denmark

**Keywords:** Atopic dermatitis, Enzyme-linked immunosorbent assay, Immunoassay, Interference, Anti-animal IgG antibodies, Heterophilic antibodies, Interleukin-33

## Abstract

**Background:**

In the search for valid biomarkers in inflammatory diseases, cytokine serum concentrations are often measured by enzyme-linked immunosorbent assay and correlated to disease activity. Interleukin-33 is a relatively newly described cytokine, which holds a promising potential as a biomarker for different diseases including atopic dermatitis. However, interfering human anti-animal IgG antibodies and heterophilic antibodies might give rise to false positive or negative results that often go unnoticed.

**Findings:**

We performed a three-step validation of commercially available and widely used human interleukin-33 enzyme-linked immunosorbent assay kit with serum samples from eight atopic dermatitis patients and five healthy controls. Through addition of unspecific animal IgG (rabbit, mouse, goat and bovine) and unspecific human IgG to the assay diluent, we disclosed false positive values in 12 out of 13 samples.

**Conclusion:**

This study show that the present human interleukin-33 enzyme-linked immunosorbent assay kit might give rise to a high prevalence of false positive values if not validated. This inaccuracy is easily eliminated with a simple set of validation steps.

## Background

Atopic dermatitis (AD) is a chronic inflammatory skin disease with high prevalence, substantial morbidity, and great effect on quality of life. Treatment is challenging and depends on previous treatment responses, side effects and disease severity (Proudfoot et al. 2013). For these reasons a reliable, consistent biomarker correlating with disease activity is needed. Interleukin-33 (IL-33) is a newly described cytokine in the context of AD (Schmitz et al. 2005). IL-33 is a nuclear cytokine from the IL-1 family constitutively expressed in epithelial barrier tissues and lymphoid organs, which plays important roles in type-2 innate immunity and atopic disease (Moussion et al. 2008). IL-33 functions as an alarmin (alarm signal) rapidly released upon cellular damage or stress (Cevikbas and Steinhoff 2012). Studies of IL-33 serum concentrations in different diseases have been undertaken also recently in AD, proposing increased concentrations compared to healthy controls (Tamagawa-Mineoka et al. 2014). We have undertaken a methodological approach to the evaluation of IL-33 in serum from AD patients.

Enzyme-linked immunosorbent assay (ELISA) is the gold standard when interrogating serum analytes but even so the challenge with interfering human anti-animal IgG antibodies (HAAA) and heterophilic antibodies can cause unnoticeable false positive or negative results (Willman et al. 2001; Kricka 1999). Recommendations have been made to face interference in immunological assays, in particular two-site (sandwich) immunoassays (Kragstrup et al. 2013). Circulating HAAAs can result from both iatrogenic and noniatrogenic causes (Kricka 1999), while heterophilic arise in healthy individuals as natural antibodies (Levinson and Miller 2002).

Several mechanisms of antibody interference are assumed (Klee 2000). One is potential bridging of the capture and signal antibody that might cause falsely high-test values. Another is that HAAAs may diminish the signal by blocking the analyte binding to capture and/or detection antibody producing incorrectly low-test values. Furthermore, antibodies against the idiotype of the original image (anti-idiotypic antibody) and therapeutic antibody that blocks the activity of the reagent capture antibody cause falsely low measurements and lastly anti-anti-idiotypic antibodies, which mimic the reagent capture antibody could also cause falsely low measurement. The presence of human anti-animal IgG antibodies against mouse and bovine that through bridging gives rise to falsely high results is presumably the biggest problems of the above mentioned and reports of extraordinary prevalence of human anti-animal IgG antibodies (95 %) in patient sera and the high correlation with false positive results (p < 0.0001) makes validation of both commercially bought and in-house ELISA kits meaningful (Andersen et al. 2004). One way of doing this is following a set of validation steps to evaluate and improve a sandwich ELISA (Kragstrup et al. 2013).

## Results

**Fig. 1:**
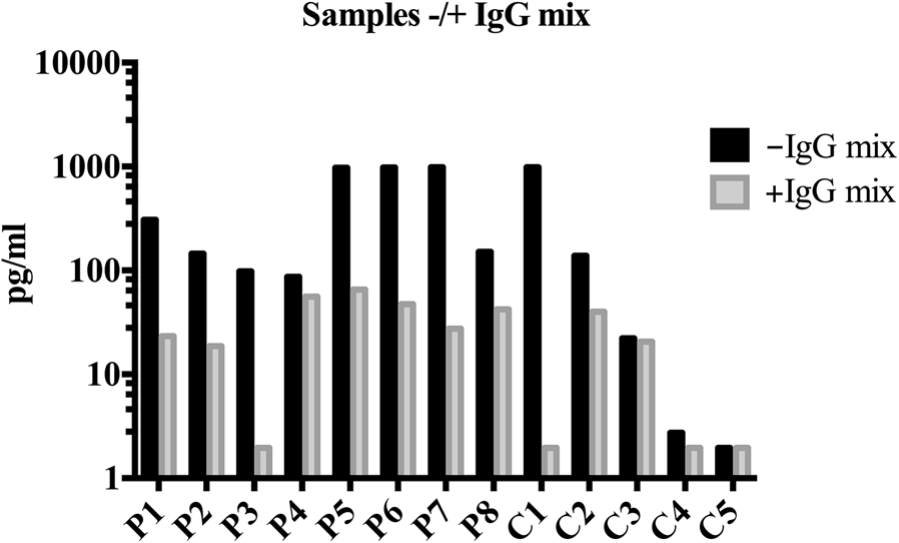
Identification and removal of false positive results by pre-incubating samples with a combination of rabbit, mouse, goat, bovine, and human immunoglobulins. Note the logarithmic y-axis. *P* patient, *C* control, *IgG* immunoglobulin G

We validated the present ELISA kit in three steps with serum samples from 8 AD patients and 5 healthy controls. We evaluated noise-to-signal ratio with two different blocking buffers. However, we did not observe any effect of additional blocking, suggesting that the blocking by the manufacturer is adequate (data not shown). Secondly, we evaluated possible false negative results by spiking samples with known concentrations of recombinant human IL-33. We did not observe any problems with false negative results (data not shown). Finally, we evaluated false positive results by adding a mix of unspecific animal IgG (rabbit, mouse, goat and bovine) and unspecific human IgG to the assay diluent. This step disclosed false positive values in 12 out of 13 samples. The serum concentrations before and after preincubation with IgG mix are shown in Fig. 1. This gave rise to an unpredictable but consistent and significant reduction of the measured values (Wilcoxon matched pairs signed rank test: p = 0.0005). The fold reduction from adding IgG mix was median 7.7; IQR 26.8; minimum 1.0; maximum 504.5. Additional details are described in the “Methods” section.

## Discussion

The evaluation of biomarkers often involves the use of sandwich ELISA techniques. Undertaking validation of any ELISA kit is important prior to main analyses as HAAA and heterophilic antibodies are commonly found in plasma from both patients and controls ultimately leading to interference and false results consequently erroneous conclusions. In this study we show that there is a high prevalence of incorrectly elevated values occurring from nonspecific HAAAs cross-linking the capture and signal antibodies of the IL-33 immunoassay by binding to isotypic determinants expressed on the Fc portions of both antibodies. We also show that this phenomenon is easily unveiled and removed with unpretentious methods of validation.

As this specific IL-33 sandwich ELISA kit was commercial manufactured the element of personal optimization is heavily restricted compared to in-house ELISA systems. Nonetheless validation makes just as much sense as with any other kit or setup and the testing of blocking agents and falsely positive/negative values is pivotal.

Two different blocking agents was tested compared to the protocol that dictated no agent at all. We showed that neither PBS with 1 % BSA nor PBS with 5 % skimmed milk decreased the noise to signal ratio thus no additional blocking was needed. We saw no false negative results in either patient or control material, though on the contrary the prevalence of false positive results was very high. This unspecific interference was eliminated by preincubation of samples with a mix of goat, rabbit, mouse, bovine and human IgG. As individual testing of each species IgG was not done we have no insight to which one might have impacted the results the most.

## Conclusion

The use of ELISA systems in serological biomarker research is both elegant and cost-effective but any kit should be subjected to a set of validation steps. This study show that the present human IL-33 ELISA kit might give rise to a high prevalence of false positive values if not validated. This inaccuracy is easily eliminated with a simple set of validation steps that could be applied to any in-house or commercially available ELISA kit.

## Methods

### Samples

Blood samples from eight patients with clinical verified AD all suffering from moderate to severe disease (SCORAD 28.0–85.5) with no other registration apart from diagnosis were used. Five healthy controls with no history of either dermal or systemic inflammatory disease matched by age and gender were included as reference. All blood samples were stabilised with EDTA before fractionation by centrifugation and plasma retrieval.

### Reagents and antibodies

The IL-33 ELISA kit used in our analyses was bought of BioLegend Inc (GeneID: 90865, Cat. No. 435908, BioLegend, San Diego, USA). It supplied anti-human IL-33 pre-coated 96-well strip microplates, IL-33 detection antibody, lyophilized human IL-33 standard, horseradish peroxidase (HRP) conjugated streptavidin, substrate solution, stop solution, assay diluent and wash buffer (20x). Rabbit, mouse, goat, and bovine IgG for blocking potential anti-animal IgG antibodies in the samples to be measured were purchased from Jackson ImmunoResearch (catalog numbers 011-000-003, 015-000-003, 005-000-003 and 001-000-003, West Grove, USA) and human immunoglobulin for blocking potential anti-human immunoglobulin antibodies in the samples were purchased from Behring (Beriglobulin, King of Prussia, USA). Buffers to block non-specific binding sites in polystyrene wells were prepared with PBS pH 7.4 and bovine serum albumin (BSA) (catalog number 12659, Calbiochem, San Diego, USA) or Skimmed milk 5 % (Part No: 1.15363.0500, EMD Millipore, Merck, Germany).

### Assay diluent

As the assay diluent came fully prepared from the manufacturer it was either used pure or mixed with a combination of 20 μg/ml mouse IgG, 20 μg/ml rabbit IgG, 20 μg/ml goat IgG, 20 μg/ml bovine IgG and 20 μg/ml heat aggregated human IgG. IgG concentrations were selected on the basis of the present literature in the field (DeForge et al. 2010; Kricka 1999; Kricka et al. 1990; Koshida et al. 2010; Kragstrup et al. 2013). All samples and standards were left to preincubate in assay diluent or assay diluent + IgG mix for 15 min at RT before addition to the plate. Samples were diluted 1:1 and 100 μl were added and the plate was incubated over night at 4 °C.

### Blocking

The need of a blocking agent was questioned as the present ELISA kit was pre-coated and the manufactures protocol does not suggest blocking of the primary antibody before application of samples. We chose three different settings to evaluate a potential need of this. We tested this in wells with blank assay diluent only and 2 h at RT with either PBS with 5 % skimmed milk or PBS with 1 % BSA or no blocking prior to adding assay diluent.

### Wash

Preceding all steps each well was washed four times with the supplied wash buffer in a 1:20 dilution with deionized water. Before adding substrate solution five washes and increased soaking time was carried out as recommended by the manufacturer.

### Capture and detection antibodies and streptavidin-HRP

All reagents were supplied in the kit and were used according to the manufacturers protocol.

### Measurement of optical density

The optical density (OD) of each well was measured within 10 min from adding stop solution. The microplate reader was set to 450 nm and wavelength correction at 570.

### Calculations and graphs

As data did not follow a Gaussian distribution a non-parametric Mann–Whitney test was used to evaluate the impact of IgG mix. Mean OD values from doublets were calculated and the mean blank value was subtracted. Adding rh IL-33 to samples from three different AD patients tested spike recovery. Recovery was calculated as OD of the spiked sample subtracted the unspiked sample, divided by the expected value and multiplied by 100 %: Recovery = (ODspiked − ODunspiked) ⁄ Expected spiked × 100 %. The standard curve was fitted with a 5-parameter logistic nonlinear regression model using the MasterPlex ReaderFit version 2.0.0.68 (MiraiBio Group of Hitachi Solutions America, Ltd, USA) and cut-off value was set at 1.95 pg/ml. Measurements below the cut-off values were assigned the cut-off value. Statistics were done in STATA version 12.0 (StataCorp, College Station, Texas, USA). Graphs were made in GraphPad Prism version 6 (GraphPad software, San Diego, USA). A *p* value of less than 0.05 was considered significant.

## Abbreviations

IL: interleukin
AD: atopic dermatitis
IgG: immunoglobulin G
ELISA: enzyme-linked immunosorbent assay
OD: optical density
SCORAD: SCORing Atopic Dermatitis
rh: recombinant human
RT: room temperature
BSA: bovine serum albumin
PBS: phosphate buffered saline
